# Lack of association of two polymorphisms of *IRF5* with Behcet’s disease

**Published:** 2009-10-07

**Authors:** Haijun Li, Peizeng Yang, Zhengxuan Jiang, Shengping Hou, Lin Xie

**Affiliations:** 1 Department of Ophthalmology, Daping Hospital, The Third Military Medical University, Chongqing, P.R. China; 2 Chongqing Key Laboratory of Ophthalmology, Chongqing Eye Institute, Chongqing, P.R. China; 3 Department of Ophthalmology, the First Affiliated Hospital, Chongqing Medical University, Chongqing, P.R. China

## Abstract

**Purpose:**

Interferon regulation factor 5 (IRF5) is a member of the IRF family of transcription factors that control the transactivation of type I interferon system-related genes as well as the expression of several other genes involved in immune response. Here, we investigated its association with Behcet’s disease (BD) in a well defined group of Chinese Han patients.

**Methods:**

A total of 152 unrelated Chinese patients with BD and 149 healthy blood donors were genotyped for *IRF5* rs2280714 and rs752637 polymorphisms. Genomic DNA was isolated from peripheral blood mononuclear cells. Genotyping of each single nucleotide polymorphism (SNPs) was performed by polymerase chain reaction restriction fragment length polymorphism (PCR-RFLP). Allele and genotype frequencies of *IRF5* rs2280714 and rs752637 polymorphisms were compared between patients and controls using a two-sided χ^2^ test.

**Results:**

The results showed no significant difference concerning the frequency of the allele of rs2280714 and rs752637 polymorphisms between BD patients and the normal controls (p=0.647 and p=0.105, respectively). The frequencies of the genotype of rs2280714 and rs752637 were not different between BD patients and the normal controls (p=0.233 and, p=0.266, respectively). Clinical manifestation stratification analysis did not show any association of *IRF5* polymorphisms with BD patients (p>0.05).

**Conclusions:**

Our study revealed that the rs2280714 and rs752637 SNPs were not associated with the susceptibility to BD. There was no association between the two polymorphisms of *IRF5* and any extraocular clinical manifestations in BD.

## Introduction

Behcet’s disease (BD) is a chronic autoimmune disease in which both genetic and environmental factors are involved. It is a common uveitis entity in China, Japan, and the Middle East countries [1]. BD is an autoimmune disease that results from damage to blood vessels throughout the body [2]. However, what triggers this reaction is unclear.

The family of interferon regulatory factors (IRF) had been implicated in providing a principal basis for host resistance against pathogens [3]. The family also plays a role in the regulation of the development of the immune system and the production of pro-inflammatory cytokine [4-7]. Studies have demonstrated that *IRF1* is associated with Behcet’s disease [8] whereas *IRF5* has been found to be associated with certain autoimmune diseases such as systemic lupus erythematosus (SLE) [9,10], inflammatory bowel disease (IBD) [11], rheumatoid arthritis (RA) [12], and multiple sclerosis (MS) [13]. Whether there is an association of *IRF5* with BD is not yet clear. Here, we examined the association of *IRF5* single polymorphisms (rs2280714 and rs752637) with BD in a Chinese Han population.

## Methods

### Patients and normal controls

A total of 152 unrelated Chinese patients with BD according to the international study group criteria [14] and 149 age-, sex-, and ethnically-matched healthy controls were included in this study. Samples from patients and controls were genotyped for *IRF5* rs2280714 and rs752637 polymorphisms after they all gave their informed consents. All study subjects were recruited from Zhongshan Ophthalmic Center (Sun Yat-sen University, Guangzhou, P.R. China), the First Affiliated Hospital, Chongqing Medical University (Chongqing, P.R. China), and Daping Hospital (the Third Military Medical University, Chongqing, P.R. China). The study was approved by the local ethics committees of the Third Military Medical University and Chongqing Medical University.

### Genomic DNA extraction and PCR

Blood samples were collected in ethylenediamine tetra acetic acid (EDTA) tubes and kept at −70 °C until required [15]. Genomic DNA was extracted from peripheral blood mononuclear cells, which was performed by conventional salting of cellular protein, an alcohol precipitation using standard proteinase K digestion, and phenol chloroform extraction procedure [16]. We amplified the target DNA in *IRF5* by polymerase chain reaction (PCR) using the relevant primers. The primers of the two remaining sites were designed using Primer Premier 5.0 software (Premier Biosoft International, Palo Alto, CA). The details of the primers and enzymes used for PCR-RFLP genotyping are presented in Table 1.

**Table 1:** The primers and enzymes used for PCR-RFLP genotyping.

**SNP**	**Forward primer sequence**	**Reverse primer sequence**	**Enzyme**
rs2280714	5′-CGTGGTCACATTGGTGATGCTC-3′	5′-GGCTCTTCTCTCCAAGGCAGACA-3′	BSMFI
rs752637	5′-AAGGTGCCCAGAAAGAAGCTTCTTAC-3′	5′-GCACTGGGAAATCACCCCTTTT-3′	BCEAI

The details of the primers and enzymes used for PCR-RFLP genotyping.

Each PCR was performed in 10 μl of solution containing 5 μl premix Taq (Takara Biotechnology Co. Ltd, Dalian, P.R. China), 10 pmol primers, and 0.2 μg of genomic DNA. The cycling profile was: initial denaturation at 94 °C for 4 min, 45 cycles consisting of 94 °C for 5 s, proper annealing temperature 63 °C for 30 s, 72 °C for 30 s, and a final extension for 4 min at 72 °C.

### Genotyping

Genotyping of each single nucleotide polymorphism (SNP) was performed by PCR restriction fragment length polymorphism (PCR–RFLP). PCR products of rs2280714 and rs752637 were respectively digested with 2 μl of BSMFI, BCEAI restriction enzymes (Fermentas, MBI, Vilnius, Lithuania) in a 10 μl reaction volume overnight. Digestion products were visualized on agarose of 2%–3% concentration and stained with Goldview^TM^ (SBS Genetech, Beijing, P.R. China). BSMFI digestion of 130 bp PCR product of AA genotype gives two fragments, the GG gives one fragment, and the AG gives three fragments. BCEAI digestion of 120 bp PCR product of AA genotype gives two fragments, the GG gives one fragment, and the AG gives three fragments. To confirm the accuracy of the method employed, randomly selected subjects (20% of all samples) were analyzed by direct sequencing (Invitrogen Biotechnology Co., Guangzhou, P.R. China). An appropriate control was included in each typing run.

### Statistical analysis

Allele and genotype frequencies of *IRF5* rs2280714 and rs752637 polymorphisms were compared between patients and controls. Statistical analysis was performed with the SPSS version 10.0 for Windows (SPSS Inc., Chicago, IL). We evaluated the frequency of genotypes and alleles in this study using the χ^2^ test. The association of rs2280714 and rs752637 with the extraocular findings was evaluated by logistic regression and statistical significance was taken when p<0.05.

## Results

The average age of the BD patients was 32.7±6.4 years and that of the healthy controls was 33.8±9.3 years. No statistical difference in the distribution of age was observed between BD patients and controls (p>0.05).

One hundred and fifty-two patients and one hundred forty-nine normal controls were genotyped for two *IRF5 *SNPs. It is worthwhile to point out that some genotypes were missing for controls for rs752637 SNPs due to insufficient amount of DNA in some samples. The results showed that there was no deviation from the Hardy–Weinberg equilibrium (HWE). The genotype and allele frequencies of the rs2280714 SNPs in BD patients were not different from those in normal controls (p=0.233 and p=0.647, respectively; Table 2). A similar result was found for rs752637 SNP versus normal controls (p=0.266 and p=0.105, respectively; Table 2). Stratification analysis according to oral ulcer, genital ulcer, hypopyon, skin lesions, and arthritis did not show any association of *IRF5* polymorphisms with these parameters (Table 3; p>0.05).

**Table 2:** Genotype and allele distributions of *IRF5* rs2280714 and rs752637 polymorphisms in BD patients and controls.

***IRF5* SNPs**	**Genotype allele**	**Patients**	**Normal controls**	**p value**	**OR (95%CI)**
rs2280714	AA	51 (56.1)	60 (54.9)	0.233	
	AG	74 (66.7)	58 (65.3)	0.233	
	GG	27 (29.3)	31 (28.7)	0.233	
	A	176 (57.9)	178 (59.7)	0.647	0.927 (0.670–1.283)
	G	128 (42.1)	120 (40.3)	0.647	1.079 (0.780–1.493)
rs752637	AA	64 (59.0)	49 (54.0)	0.266	
	AG	68 (68.4)	63 (62.6)	0.266	
	GG	20 (24.5)	27 (22.5)	0.266	
	A	196 (64.5)	161 (57.9)	0.105	1.319 (0.944–1.843)
	G	108 (35.5)	117 (42.1)	0.105	0.758 (0.543–1.059)

BD=Behcet’s disease, OR=odds ratio, 95% CI=95% confidence interval. The genotype and allele distributions of IRF5 rs2280714 and rs752637 polymorphisms in BD patients and controls. No significant differences of the genotype and allele distributions were found between patients and controls.

**Table 3:** The clinical manifestations of BD patients.

**Clinical manifestations**	**N**	**Percentage**
Uveitis	152	1%
Oral ulcer	127	0.836%
Genital ulcer	64	0.421%
Hypopyon	41	0.27%
Skin lesions	84	0.553%
Arthritis	43	0.283%

N=number of patients; BD=Behcet’s disease. This table summarizes the proportion of clinical manifestations in relation to the BD patients

## Discussion

It has been demonstrated that both genetic and environmental factors are involved in the development of BD [17]. In this study, we investigated the association of *IRF5* polymorphisms with BD in the Chinese Han population. Our study showed that rs2280714 and rs752637 were not associated with BD. There was no association between the polymorphisms of *IRF5* with any extraocular clinical manifestations.

BD is a common multisystem inflammation disorder characterized by recurrent oral and genital ulceration, multiple skin lesions and uveitis [18,19]. It has a distinctive geographical distribution. *IRF5* has been shown to be strongly associated with certain autoimmune diseases in different ethnic populations [10,11,20,21] and to play a critical role in the pathogenesis of autoimmunity [22]. Therefore, this gene has been considered as a general susceptible gene for autoimmune diseases. However, the genetic factors associated with this disease in Chinese Han patients have been scarcely studied. In this study, we focused on the association of two SNPs of *IRF5* with BD in the Chinese patients. As population stratification may lead to a false-positive result in a case-control study, we strictly selected the BD patients and controls with Chinese Han nationality. To validate the result of genotyping by PCR-RFLP, 20% of the samples were directly sequenced.

Unfortunately, in this study, we did not find any association of rs2280714 and rs752637 SNPs with BD. A similar result was observed after stratification analysis based on extraocular features. These results are not consistent with those seen in other autoimmune diseases reported in European and some other Asian populations [10,12,20,21]. This could be explained by the fact that the etiology and pathogenesis of BD may be different from those of the other autoimmune diseases [2,18,19]. The other possibility could be that Chinese Han people may have a different genetic background from other ethnic populations [2,19]. Additionally, association of some other *IRF5* SNPs should be tested in BD.

Lee and coworkers found the association of *IRF1* polymorphism with Behcet’s disease in Koreans [8]. Here, we failed to find the association of *IRF5* with BD. The inconsistent result can be explained by the different immune function of IRF1 from IRF5. Both of them interact with MyD88, an adaptor for multiple innate immune receptors, but induce a distinctively different set of pro-inflammatory cytokines. These results suggest that IRF1 and IRF5 play different roles in the production of pro-inflammatory cytokine and the regulation of the development immune system, that is to say, different mechanisms may be induced by IRF1 than by IRF5 in the development of BD disease.

In conclusion, our study revealed that the rs2280714 and rs752637 SNPs were not associated with the susceptibility to BD. There was no association between the two polymorphisms of *IRF5* with any clinical manifestations. Further studies are needed to investigate the association of other SNPs in *IRF5* with BD.
